# Voltage-Gated Ion Channel Compensatory Effect in DEE: Implications for Future Therapies

**DOI:** 10.3390/cells13211763

**Published:** 2024-10-24

**Authors:** Khadijeh Shabani, Johannes Krupp, Emilie Lemesre, Nicolas Lévy, Helene Tran

**Affiliations:** Institut de Recherches Servier, Rue Francis Perrin, 91190 Gif-sur-Yvette, France; johannes.krupp@servier.com (J.K.); emilie.lemesre@servier.com (E.L.); nicolas.levy@servier.com (N.L.)

**Keywords:** Developmental and Epileptic Encephalopathy (DEE), ion channels, compensatory effect

## Abstract

Developmental and Epileptic Encephalopathies (DEEs) represent a clinically and genetically heterogeneous group of rare and severe epilepsies. DEEs commonly begin early in infancy with frequent seizures of various types associated with intellectual disability and leading to a neurodevelopmental delay or regression. Disease-causing genomic variants have been identified in numerous genes and are implicated in over 100 types of DEEs. In this context, genes encoding voltage-gated ion channels (VGCs) play a significant role, and part of the large phenotypic variability observed in DEE patients carrying VGC mutations could be explained by the presence of genetic modifier alleles that can compensate for these mutations. This review will focus on the current knowledge of the compensatory effect of DEE-associated voltage-gated ion channels and their therapeutic implications in DEE. We will enter into detailed considerations regarding the sodium channels SCN1A, SCN2A, and SCN8A; the potassium channels KCNA1, KCNQ2, and KCNT1; and the calcium channels CACNA1A and CACNA1G.

## 1. Introduction

Developmental and Epileptic Encephalopathies (DEEs) refer to a group of neurologic disorders that are clinically and genetically heterogeneous. They are characterized by intractable epileptic seizures starting in infancy, accompanied by developmental delay and cognitive impairments, difficulties with motor control, and a high susceptibility to sudden unexpected death in epilepsy (SUDEP) [1,2]. Most DEEs are rare genetically determined disorders, but due to their genetic heterogeneity, their precise and specific epidemiology remains difficult to evaluate, while the overall annual incidence of single-gene epilepsies has been estimated at around 1 per 2100 live births [3,4]. The vast majority of DEE disease-causing genomic variants arise *de novo*, and a few are inherited from an unaffected mosaic parent [5,6]. These mutations include gain or loss of function (GOF/LOF) in genes that control neuronal excitability, synapse function, and brain development. Several types of mutations have been described, including small insertions, deletions, frameshifts, nonsense, missense, splice-site mutations, and full exon deletions or duplications [7,8]. Voltage-gated ion channels (VGCs) are transmembrane proteins mediating ion fluxes across membranes. They include sodium, potassium, calcium, and chloride channels, coordinating the flux of cationic and anionic currents to regulate the electrical features of neurons. They are the core to the regulation of membrane resting potential, generation, and conduction of action potential (AP), modulation of cell excitability, and neurotransmitter release. While they are broadly distributed throughout the brain, their expression varies across different regions of the brain and is differentially regulated within dendrites and axons. This tight distribution and regulation allow them to generate action potential firing patterns and integrate synaptic inputs properly [9]. This review will examine the latest understanding of the compensatory effects of specific voltage-gated ion channels associated with DEE and their therapeutic implications. The ion channels under consideration include three sodium channels, SCN1A, SCN2A, and SCN8A; three potassium channels, KCNA1, KCNQ2, and KCNT1; and two calcium channels, CACNA1A and CACNA1G (the summary of these channels is provided in Table 1). We will begin with an introduction to these channels and their regulation pattern throughout brain development, followed by an exploration of their compensatory effects as a potential therapeutic strategy in DEE.

## 2. Voltage-Gated Sodium Channels

Voltage-gated sodium channels (Nav) **in** the brain include one large, pore-forming α-subunit and two smaller β-subunits [10]. The α-subunit has roughly 2000 amino acids and includes four homologous domains (DI to DIV), each containing six highly conserved transmembrane segments (S1 to S6). The α-subunit is responsible for conducting ion currents, whereas the β-subunits control trafficking and some electrophysiological properties of the α-subunit [11,12]. *SCN1A* (encoding Nav1.1) is highly expressed in the CNS, and its expression steadily increases from 4–5 months of age [13]. At the cellular level, it is predominantly expressed in the GABAergic interneurons (particularly in parvalbumin neurons), regulating neuronal excitability, and at the subcellular level, it is found predominantly in the axon initiation segment (AIS) of neurons, where it is thought to control setting the action potential initiation threshold [14]. SCN1A is the most common genetic cause for DEE, with GOF or LOF mutations that scatter across almost all regions of the protein [15]. More than 1700 disease-causing variants have been reported for SCN1A, and more than 1500 of them are related to epileptic disease (SCN1A mutation database; caae.org.cn). Dravet syndrome (DEE6A) is the most common DEE due to LOF mutations (missense, nonsense, or truncations/deletions) in SCN1A. Most patients (~90%) have *de novo* heterozygous mutations, whereas 5–10% carry inherited autosomal dominant mutations from an asymptomatic or mildly affected parent [11]. The incidence of DEE6A is estimated at 1 in 20,900 in the US population [16], 1 in 22,000 in the Danish population [17], and 1 in 40,900 in the UK population [18], where the average age at the onset of a seizure is 6 months. Development is commonly normal during the first year, but many patients develop cognitive, intellectual, and motor co-morbidities during the second year of life [19]. The severity of the phenotype varies even among family members with the same pathogenic variant [20]. Similarly, the lethal seizure shows incomplete penetrance in the mouse models of Dravet syndrome, with 50% of Scn1a^+/−^ mice remaining unaffected. This may be explained by compensatory upregulation of other sodium channels to compensate for the LOF of SCN1A [21,22]. *SCN2A* (encoding Nav1.2) is widely expressed throughout the human central nervous system but not in peripheral tissues [23]. It is highly expressed in the fetal brain with a close developmental correlation with SCN8A, where it peaks during early development and is successively replaced by SCN8A during the first month of life [24]. It is expressed predominantly, but not exclusively, in excitatory glutamatergic neurons, where it is more localized in proximal axon initial segments (AIS) and involved in the initiation and propagation of action potential [25]. It is also localized to the soma and dendrites of pyramidal neurons [26]. It is transcribed in neonatal and adult isoforms, each with different characteristics, where the neonatal splice isoform of SCN2A is less excitable than that of adults. This reduced excitability in the neonatal isoform provides a protective role against seizures. In contrast, the adult Scn2a isoform in mice is associated with increased seizure susceptibility [27]. More than 100 mutations have been reported for this gene, where GOF mutations are related to epilepsy due to neuronal hyperexcitability, and LOF mutations are related to autism and the intellectual disability phenotype. Early-onset (EO) and late-onset (LO) forms of the disorder have been reported. In EO form, seizure onset occurs before 3 months of age, and it is related to GOF mutations. In LO form, seizure onset occurs after 3 months of age, and it is linked with partial or complete LOF of SCN2A, including missense, frameshift, nonsense, and splice site mutations [11,24,28]. *SCN8A* (encoding Nav1.6) is expressed in the central and peripheral nervous systems ubiquitously. At the cellular level, it is expressed predominantly in excitatory neurons, especially in cerebellar granule cells, as well as pyramidal and granule cells of the hippocampus. Nav1.6 is expressed prenatally, and the expression begins to increase shortly after birth, reaching its highest level during the first years of life. At the subcellular level, it is widely expressed in the distal AIS and nodes of Ranvier of myelinated axons. The distal AIS is where the action potentials are triggered, and Nav1.6 exhibits a 40-fold higher concentration of the AIS than the soma and proximal dendrites [29]. SCN8A plays a crucial role in initiating action potentials at the distal AIS, and the absence of Nav1.6 leads to an elevated threshold for action potential initiation [30,31]. More than 200 mutations have been reported in SCN8A, where these mutations can manifest as both GOF and LOF, with the majority of epilepsy-linked variants being de novo GOF missense mutations [32,33]. It has been shown that overexpression of Nav1.6 in the AIS results in an increase in spontaneous and repetitive firing [25,34], a possible rationale for why epilepsy-related mutations in SCN8A are mainly GOF with an effect on the action potential threshold.

## 3. Voltage-Gated Potassium Channels

Voltage-gated potassium channels (Kv) are the largest ion channel group with substantial expression in CNS. They are tetramers composed of four pore-forming α-subunits and modulatory β-subunits. Each α-subunit contains six transmembrane segments (S1–S6) where S1–S4 form the voltage-sensor domain, and the S5–S6 linker form the pore region. They control resting membrane potential (RMP) by producing outward K+ currents followed by limiting neuronal excitability [35,36,37]. *KCNA1* (encodes Kv1.1) is widely expressed throughout the central nervous system, with particularly high levels of expression in the hippocampus [38]. It assembles as a heteromer with KCNA2 (encodes Kv1.2) to regulate outward potassium currents, orchestrate action potentials, and finely tune neurotransmitter release. KCNA1 is predominantly localized in the axon initial segment, axon preterminal, and the juxta-paranodal (JPN) domain besides the nodes of Ranvier [39]. In the neocortex, they are located at the AIS of fast-spiking neocortical GABAergic interneurons and regulate their excitability [40]. In the cerebellum, they are at the terminal of basket cells, where they suppress the hyperexcitability of Purkinje cells by promoting GABA release [41]. Mutations in the KCNA1 gene induce LOF defects and change the biophysical properties of heteromeric Kv1 channels, causing epilepsy or Episodic Ataxia Type 1 (EA1) or a mixed phenotype [35]. *KCNQ2* (encoding Kv7.2) acts as homo-tetramers or hetero-tetramers with KCNQ3 (Kv7.3) to mediate the M-current, a slow, non-inactivating K+ current that regulates membrane potential at the subthreshold voltage range, thus limiting action potential initiation and repetitive firing frequency. It is widely expressed in the brain and enriched in the AIS and nodes of Ranvier in pyramidal neurons [39,42]. KCNQ2 variants might cause either benign epilepsies or DEE, depending on the protein domain affected by the mutation. The majority of KCNQ2 and KCNQ3 variants are LOF. HoweverGOF, some de novo GOF variants are also related to DEE [43]. *KCNT1* encodes the sodium-activated potassium channel KNa1.1 (also known as Slo2.2 or Slack), which, in collaboration with KNa1.2 (encoded by KCNT2), mediates a delayed outward rectifying K+ current, thus modulating neuronal excitability and adaptability in response to high-frequency stimulation [44]. Slack channels are widely expressed in different regions of the brain, including the cortex, hippocampus, thalamus, olfactory bulb, midbrain cerebellum, and brainstem [45,46]. The activity of KCNT1 is, like that of KCNT2, weakly voltage-dependent and can be modulated by the intracellular sodium level. Mutation in KCNT1 shows a strong GOF effect, which results in augmented channel activity and an increase in potassium current up to 22-fold compared to wild-type channels [47,48]. KCNT1 channels are expressed in both inhibitory and excitatory neurons and are predominantly localized in some dendritic regions of the neurons. GOF variants impair excitability and AP generation of inhibitory interneurons but not excitatory neurons, leading to the loss of inhibitory regulation and seizure susceptibility [49,50,51].

## 4. Voltage-Gated Calcium Channels

Voltage-gated calcium (Cav) channels are hetero-multimers proteins that control calcium influx in response to membrane depolarization and regulate neurotransmitter release, intracellular signaling, and gene expression in the CNS. They consist of the pore-forming principal α subunit, which allows calcium entry in response to depolarization, and auxiliary subunits, including β, α2δ, and γ, that regulate channel localization and function. The α subunits are encoded by 10 different genes and contain four domains (DI-DIV), each with six transmembrane segments (S1 to S6), where the S5 and S6 transmembrane segments line the inner pore of the channel [10,52]. *CACNA1A* encodes the α subunit of high-threshold Cav2.1 P/Q-type voltage-gated calcium channel and regulates neurotransmission in excitatory and inhibitory synapses. Around 1700 CACNA1A variants have been reported, and over 400 of them are pathogenic or likely pathogenic with limited or no clinical or functional data [53]. Mutations in CACNA1A, whether inherited or occurring de novo, have been linked to severe early infantile epileptic encephalopathy in children. This condition manifests due to a specific loss of Cav2.1 channels in parvalbumin-positive GABAergic interneurons located in the cortex and hippocampus. This selective loss leads to impaired GABA release, which in turn triggers seizures [54]. *CACNA1G* encodes Cav3.1, a low threshold T-type voltage-gated calcium channel conducting transient calcium currents in response to small membrane depolarizations. It is expressed in various areas of the CNS, showing particularly high levels in Purkinje neurons and the deep nuclei of the cerebellum [55]. In mouse adult brains, Cav3.1 is localized in the cell bodies and dendritic shafts of interneurons in the cerebellum and hippocampus [56]. Missense variants of CACNA1G have been reported in DEE patients with a wide variety of seizure types. These variants typically result in a GOF effect by facilitating channel activation and prolonging the channel’s inactivation time. In contrast, the LOF of CACNA1G is protective against absence seizures, although the mechanistic details are unclear [57,58,59,60].

## 5. Developmental Regulation of Selected Voltage-Gated Ion Channels

**Figure 1:**
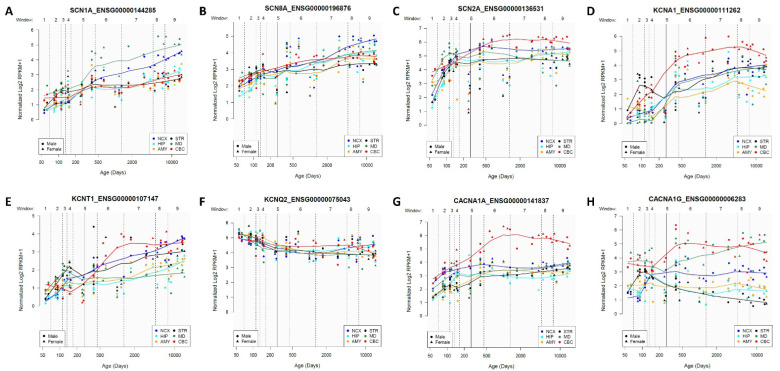
Expression profile of the selected VGCs throughout the human lifespan. Among the sodium channels, the expression of (**A**) SCN1A is lowest prenatally, while the expression of (**B**) SCN8A and (**C**) SCN2A is slightly higher, followed by a rise in all three channels expression towards adulthood. Among potassium channels, (**D**) KCNA1 and (**E**) KCNT1 show a lower level of expression during early development and increase as development progresses. On the contrary, (**F**) KCNQ2 shows the highest expression prenatally, followed by a slight decrease postnatally. (**G**) CACNA1A has a higher postnatal expression, with the highest level in the cerebellar cortex. (**H**) CACNA1G shows fluctuation within the brain regions, with the highest expression in the cerebellar cortex. Nine windows (W) include fatal development (W1 to W4), birth (W5), infancy and childhood (W6 to W7), adolescence, and adulthood (W8 to W9) in different regions of the brain, including the neocortex (NCX), hippocampus (HIP), amygdala (AMY), striatum (STR), mediodorsal nucleus of the thalamus (MD), and cerebellar cortex (CBC). The graphs are extracted from the *PsychNode dataset*; Li et al., 2018 (*PsychEncode*) [61].

Voltage-gated ion channels are developmentally regulated, where the prenatal and postnatal expression patterns differ temporally and spatially. Using the *PsychNode dataset* [61] (*PsychEncode*; showing the expression pattern of each gene from postconception week (PCW) 5 to 64 years old), we plotted the expression patterns of aforementioned channels (SCN1A, SCN2A, SCN8A, KCNA1, KCNQ2, KCNT1, CACNA1A, and CANA1G) in different regions of the human brain. The expression of SCN1A is lowest prenatally among the three sodium channels and rises progressively towards adult age (Figure 1A). Prenatal expression of SCN8A is slightly higher than that of SCN1A but follows the same trend as SCN1A (Figure 1B). SCN2A is expressed more prenatally than the two others, shows an increasing pattern until PCW 12, and remains steady across all ages (Figure 1C). Notably, the temporal expression pattern of each of these three genes aligns with the onset of the epilepsy phenotype, meaning channels that are enriched prenatally exhibit seizures earlier than those that are enriched postnatally [62]. Therefore, seizures typically begin earlier in patients with missense variants of *SCN2A*, usually around 2 weeks after birth, while they start at around 4 months and 6 months in patients carrying missense in *SCN8A* and SCN1A respectively [28]. Additionally, in utero seizures and dentate-olivary dysplasia have been reported due to mutations in *SCN2A*, suggesting a role in early neuronal development [63]. SCN2A and SCN8A show a close developmental correlation where SCN2A expression peaks in early development and will gradually be replaced at the AIS and nodes of Ranvier by SCN8A during the first month of life [64]. Milder forms of *SCN2A* related epilepsy (Benign Familial Infantile Seizures (BFISs)) are a good example of this correlation. The disease is characterized by seizures onset before 12 months, which will be resolved by 2 years of age, probably because SCN2A dysfunction is compensated by the emergence of SCN8A channels, which have a lower threshold for generation of action potential compared to SCN2A. Similarly, Scn2a could compensate for Scn8a in mouse neurons lacking Nav1.6, and that explains why seizures start later with *SCN8A* mutations compared to *SCN2A* mutations [24,28,64]. This developmental replacement of SCN2A by SCN8A is brain region dependent; for example, Nav1.2 expression continues throughout development in the cerebellum and is co-expressed with Nav1.6 in unmyelinated axons of excitatory granule cells, which is opposite to the developmental replacement of Nav1.2 in cortical myelinated axons [24,65,66]. The expression pattern of potassium and calcium channels is also highly regulated during brain development, depending on the stage of development and the brain region [67]. KCNA1 (Figure 1D) and KCNT1 (Figure 1E) show a lower level of expression during the early developmental phases and increase as brain development progresses. On the contrary, KCNQ2 shows an opposite pattern, with the highest expression prenatally in immature neurons, followed by a decrease as neuronal maturation progresses and synaptic connections form (Figure 1F). CACNA1A is initially expressed in several brain regions, including the cerebellum and cortex, and its expression gradually increases across most brain areas over time, with the highest levels observed in the cerebellar cortex (CBC), as illustrated in Figure 1G. The expression of CACNA1G shows distinct fluctuations among brain regions. Prenatally, higher expression levels are observed in the CBC and mediodorsal nucleus of the thalamus (MD) compared to other regions, followed by a postnatal increase. Conversely, it reaches a peak during mid-gestation in the striatum (STR) and neocortex (NCX), after which it declines in the striatum but persists in the neocortex, as illustrated in Figure 1H.

## 6. Compensatory Effect of Voltage-Gated Ion Channels in DEE

The homeostatic control of intrinsic excitability refers to the fact that a neuron that is dissected out from *in vivo* and transferred *in vitro* rebalances its ion channel surface expression and restores intrinsic firing properties over a period of time [68]. Ion channels are the key to keeping the homeostatic state of brain activity, and neurons continuously adjust the expression and functionality of the ion channels. For example, neurons downscale their intrinsic excitability while exposed to chronic excitotoxicity through homeostatic synaptic downscaling, a negative feedback response to chronically elevated network activity to decrease the firing rate of neurons, and/or using the compensatory effects of ion channels on each other’s functions. The compensatory effects of ion channels indicates that they can act as genetic modifiers, meaning they have the ability to affect phenotypic and/or molecular expression of each other, leading to changes in cellular behavior or physiology [69,70,71,72]. This idea—which will be discussed in detail below—is supported by several studies showing that manipulation of an ion channel gene leads to compensatory expression of other ion channels, meaning that ion channel expression is not a fixed parameter tied to cell fate. Instead, a given cell type can maintain its unique firing properties by varying combinations of ion channels.

Different groups have reported the compensatory effect of ion channels, highlighting the co-existence of variants that modulate the clinical phenotypes of the disease. Bergren et al., using the mouse model Scn2a*^Q54^*, showed that clinical severity is influenced by genetic background. This finding suggests the presence of dominant modifier alleles that may account for the phenotypic variability in patients with mutations in the SCN2A gene [73]. Martin et al. showed that double heterozygous mice for both Scn1a and Scn8a had a higher threshold for drug-induced seizures and outlived heterozygous Scn1a only mice, suggesting Scn8a acts as a genetic modifier in Scn1a mouse model of Dravet syndrome [74]. This finding is supported by other groups reporting that a reduction of Nav1.6 channels might increase survival and seizure resistance in Scn1a mutant mice [74,75,76]. Hawkins et al., using a combination of human epilepsy-related mutations, demonstrate that variants in Scn2a, Scn8a, and Kcnq2 can noticeably influence the phenotype of mice carrying the Scn1a-R1648H mutation. Combining Scn1a^RH/+^ with either Scn2a*^Q54^* or Kcnq2^VM/+^ causes early-onset generalized seizures and juvenile lethality in double heterozygous mice. In contrast, Scn8a mutants exhibit a protective effect by increasing resistance to induced seizures; combining Scn1a^RH/+^ and Scn8a^med-jo^ alleles restores normal thresholds to flurothyl-induced seizures in Scn1a^RH/+^ heterozygotes and improved survival of Scn1a^RH/RH^ homozygotes [76]. Moreover, a 25–50% reduction in the Scn8a transcript can delay seizure onset and lethality not only in mouse models of Scn8a encephalopathy but also in the mouse model of Dravet syndrome [77]. While a reduction in Nav1.6 can raise the seizure threshold in the animal model of Dravet syndrome, other sodium channel Scn9A can lower the seizure threshold and worsen the disease compared with Scn1a-only mutants [78,79]. Moreover, administering GS967 -a selective blocker of Nav1.6- reduces spontaneous seizures and prolongs the survival of heterozygous mice of N1768D Scn8a and Scn1a+/− Dravet syndrome [80]. The close developmental correlation between SCN2A and SCN8A -that was discussed earlier- is reinforced by their observed compensatory effects, suggesting a dynamic interplay during development. Nav1.2 was able to compensate for neurons lacking Nav1.6 in Scn8a^med^ (Nav1.6 null) mice [64], and GS967 is able to suppress seizure frequency by >90% and improve the survival of Scn2a*^Q54^* mice [81]. Therefore, the ability to rescue each other’s function shows how closely SCN2A and SCN8A are related.

**Figure 2:**
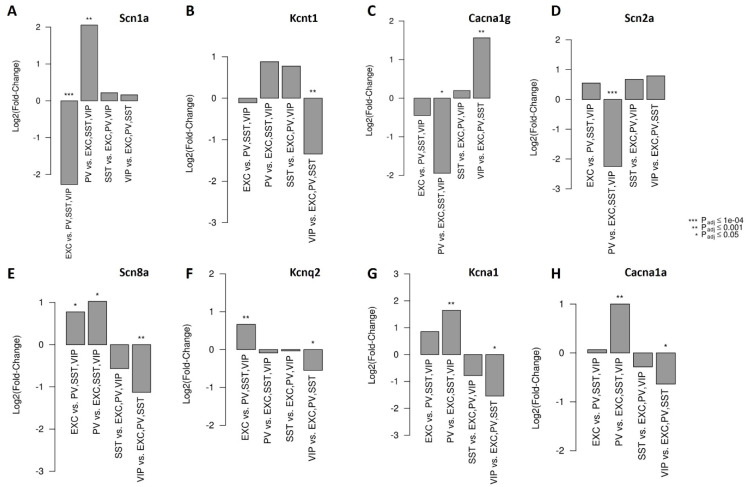
Differential expression profile of the selected voltage-gated ion channels in inhibitory (PV, SST, VIP) vs excitatory (EXC) neurons in adult mice cortex. (**A**) Scn1a, (**B**) Kcnt1, and (**C**) Cacna1g show enrichment in inhibitory neurons, whereas (**D**) Scn2a, (**E**) Scn8a, (**F**) Kcnq2, (**G**) Kcn1a, and (**H**) Cacna1a display higher enrichment in excitatory neurons (all the graphs extracted from http://research-pub.gene.com/NeuronSubtypeTranscriptomes (accessed on 8 September 2024); Huntley et al., 2020 [91]).

**Figure 3:**
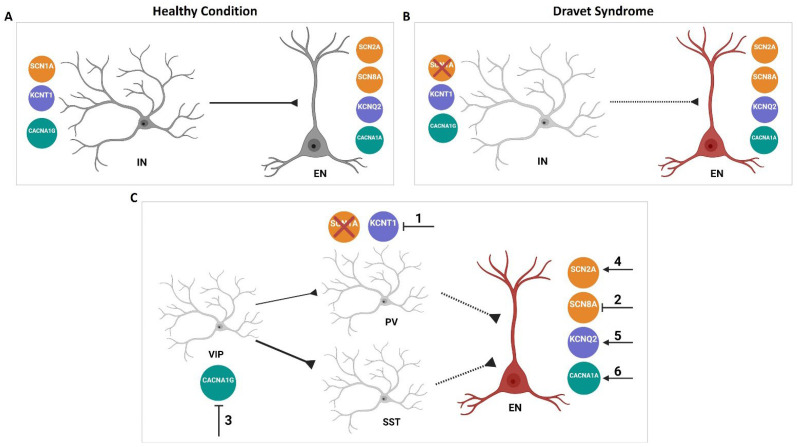
Model of the compensatory effect of certain VGCs in Dravet syndrome. (**A**) Enrichment pattern of selected voltage-gated ion channels in excitatory neurons (EN) and inhibitory neurons (IN) in healthy conditions. (**B**) SCN1A LOF in Dravet syndrome causes hypo-excitability in inhibitory neurons, leading to hyper-excitability of excitatory neurons. (**C**) Manipulating the excitation/inhibition (E/I) ratio by targeting different genes in Dravet syndrome. Inhibiting KCNT1 (Scenario 1), SCN8A (Scenario 2), and CACNA1G (Scenario 3) improves the seizure phenotype, probably by bringing the E/I ratio closer to its optimal level. In contrast, inducing SCN2A (Scenario 4), KCNQ2 (Scenario 5), and CACNA1A (Scenario 6) worsens the phenotype due to increased excitability in an already hyperexcitable network. Sodium, potassium, and calcium channels are shown in orange, purple, and green, respectively. PV, SST, and VIP show inhibitory neurons, and EN refers to excitatory neurons. The model is generated using BioRender (BioRender: Scientific Image and Illustration Software, www.biorender.com).

This compensatory mechanism is not only observed in ion channels within the same family (i.e., sodium channels) but it has also been reported in ion channels from different families (i.e., sodium vs. potassium or calcium channels). For example, Scn1a+/− mice with decreased Cacna1g (Cav3.1) expression showed improved survival and reduced spontaneous seizure frequency, suggesting CACNA1G as a possible genetic modifier and a potential drug target for Dravet syndrome patients [82]. In contrast to the ameliorating effect of Cacna1g on the mouse model of Dravet syndrome, CACNA1A has a worsening effect on Dravet syndrome, meaning subjects carrying both SCN1A and CACNA1A variants develop absence seizures earlier and more frequently than patients with only SCN1A mutations [83]. Moreover, targeting Scn8a in both Kcna1 and Kcnq2 mutant mice increases survival in both conditions as well as reducing the seizure frequency in Kcnq2 mutant mice [84]. In addition, targeting Kcnt1 transcript in Scn8a mutant mice doubled their lifespan and extended the survival of Scn1a mutant mice, suggesting KCNT1 could be a therapeutic target for the treatment of SCN1A and SCN8A epilepsy [85]. Mishra et al. showed that heterozygous deletion of the Scn2a gene (Scn2a+/−) acts as a protective genetic modifier in the SUDEP-prone Kcna1^−/−^ mice. Scn2a+/−; Kcna1−/− mice exhibit a two-fold increase in survival and significantly decreased seizure durations compared with Kcna1−/− mice [86]. Moreover, double mutant mice carrying the Scn2a*^Q54^* mutation together with either of the Kcnq2 mutations (Kcnq2*^VM^* or Kcnq2*^Szt1^*) exhibited severe epilepsy with early onset seizures and lethality by 3 weeks of age [87]. In addition, Cacna1g acts as a modifier gene and influences the severity of the Scn2a*^Q54^* phenotype. Elevated Cacna1g expression causes increased spontaneous seizure frequency in Scn2a*^Q54^* mice, whereas a decrease in the expression of Cacna1g results in decreased seizure frequency [88]. These results demonstrate that genetic interactions can impact disease severity and support the hypothesis that genetic modifiers contribute to the clinical variability observed in the DEE. This, in turn, suggests that targeting a non-causal ion channel could be an alternative useful drug target for a disease-causal mutation in another ion channel. Several questions arise in this context: how can these functional interactions be explained? Why do some of the interactions worsen the epilepsy phenotype while others are protective? Can we predict which ion channel will improve or worsen the phenotype? To answer these questions, it is important to know that an imbalanced excitation/inhibition (E/I) ratio is the root of all types of epilepsies. The neural circuit continuously coordinates their excitatory and inhibitory inputs through two types of neurons: glutamatergic excitatory neurons and GABAergic inhibitory interneurons. Enhanced excitation in excitatory neurons and/or inhibition in inhibitory interneurons leads to an impaired E/I, causing circuit hyperactivity and resulting in epilepsy [89,90]. Therefore, the compensatory effects aim to maintain brain homeostasis by restoring the excitatory/inhibitory balance. To achieve this, understanding the differential expression profiles of each channel across various neuronal types is a must. Huntley et al. explored the transcriptomics of three major classes of inhibitory neurons, including parvalbumin (PV), somatostatin (SST), and vasoactive intestinal peptide (VIP) versus excitatory neurons within the adult mouse cortex. Their findings showed that Scn1a, Kcnt1, and Cacna1g are enriched in inhibitory neurons (Figure 2A–C), whereas Scn2a, Scn8a, Kcnq2, Kcn1a, and Cacna1a display higher enrichment in excitatory neurons compared to their inhibitory counterparts (Figure 2D–H) [91]. Consistent with that, Du et al., using single-cell RNA seq transcriptomic data from humans and mice brains, show that SCN1A is predominantly expressed in inhibitory neurons, while SCN2A and SCN8A are profoundly expressed in excitatory neurons [92]. We hypothesize that a detailed understanding of the expression patterns of these selected VGCs, along with their enrichment in specific cell types, could shed light on their potential compensatory interactions. Therefore, we present a model in Figure 3, where we speculate on the protective or worsening effects of certain VGCs in a mouse model of Dravet syndrome as an example. As previously mentioned, the downregulation of SCN8A [74,75,76,80], KCNT1 [85], and CACNA1G [82] are protective in the DS mouse model. In contrast, variants in CACNA1A [83], Scn2a*^Q54^*, and Kcnq2^VM^ [76] worsen the phenotype. How can these opposite effects be explained? In the case of SCN1A LOF, hypo-excitability occurs in inhibitory neurons, leading to hyper-excitability in excitatory neurons and an impaired E/I ratio compared to a healthy condition (Figure 3A,B). To restore the E/I ratio, either excitatory neurons need to be less excitable or inhibitory neurons need to be more excitable. Targeting KCNT1 (Scenario 1 in Figure 3C), enriched in inhibitory neurons, could decrease potassium outward current in PV neurons, normalizing their intrinsic excitability and thus providing a protective effect. Downregulation of SCN8A (Scenario 2 in Figure 3C), which is enriched in excitatory neurons, could reduce the sodium entry to excitatory neurons and probably their excitability and may explain its protective effect.

The inhibition of pyramidal neurons by PV and SST interneurons is widely acknowledged, and a decrease in their function has been observed in animal models of epilepsy [93,94]. In contrast, VIP interneurons inhibit SST interneurons and, to a lesser extent, PV interneurons, resulting in reduced inhibition of pyramidal neurons [95,96]. Consistent with that, the reduced function of VIP interneurons can oppose seizure induction in animal models [97]. Furthermore, the downregulation of CACNA1G (Scenario 3 in Figure 3C), which is enriched in VIP neurons, could lead to the disinhibition of PV and SST neurons. This would probably enhance the inhibitory effects of PV and SST neurons on excitatory neurons, thereby bringing the excitation/inhibition (E/I) ratio closer to its optimal level. In contrast, the gain of function in SCN2A, KCNQ2, and certain CACNA1A variants (as shown in scenarios 4 to 6 in Figure 3C), which are all enriched in excitatory neurons, increase excitability in an already hyperexcitable network caused by the loss of SCN1A function, thereby worsening the phenotype. We acknowledge the intricate world of ion channels and the fact that it cannot be explained by a simple model. However, the protective or worsening effect of other DEE-causing mutations could be explored using this model. In conclusion, understanding the functional interaction of ion channels and subsequently modulating the balance between excitatory and inhibitory signals may serve as a-if not the key-strategic approach for targeted therapy of DEE. This means that the compensatory effects observed in various studies may open new horizons for leveraging diverse functional interactions as a promising therapeutic tool. This approach has the potential to develop single medications with the capability to target a broader population of patients.

## 7. Potential Therapeutic Strategy for DEE

Developmental Epileptic Encephalopathies belong to the group of drug-resistant epilepsy where the majority of patients experience persistent or even worsening seizures despite treatment with common antiepileptic drugs (AEDs). This challenging aspect emphasizes the need for innovative therapeutic strategies aimed at the precision treatment of DEEs. Therefore, the development of new therapeutic tools, including Antisense Oligonucleotide (ASO), has been of great interest in recent years. ASOs are synthetic, single-stranded nucleic acids that contain 10 to 30 nucleotides that modulate gene expression at the RNA level. This modulation is achieved either by RNase H1-dependent degradation of the mRNA or steric block of ribosomal activity in order to regulate RNA splicing [98,99]. ASOs have been approved as a therapeutic option for neuromuscular conditions, including spinal muscular atrophy [100], Duchenne’s muscular dystrophy [101], and amyotrophic lateral sclerosis (ALS) [102]. There have been several attempts using ASO to target the DEE-associated gene SCN1A. For example, Hsiao et al., using an ASO blocking SCN1ANAT, were able to induce upregulation of SCN1A both in vitro and in vivo in the brain of a Dravet knock-in mouse model and non-human primate. SCN1ANAT is part of an endogenous RNA-based regulatory mechanism called natural antisense transcripts (NATs), which downregulate the expression of the sense strand of the target gene. This approach led to the upregulation of healthy Scn1a and significant improvements in seizure phenotype and excitability of hippocampal interneurons in postnatal Dravet mice [103]. In another example, an ASO was used to prevent the inclusion of a poison exon (PE), thereby increasing the abundance of the full-length transcript. This approach also rescued seizures in a mouse model of Dravet syndrome and is currently in a clinical trial [104,105]. In addition to ASO, Hm1a, which is a spider venom peptide, serves as a good example of a precision medicine approach for Dravet syndrome. It has been shown that it selectively potentiates Nav1.1 without affecting other sodium or potassium channels. Richard et al. showed that intracerebroventricular infusion of Hm1a rescues seizures and premature death in a Dravet syndrome mouse model. This selective activation of Nav1.1 by Hm1a restores the function of inhibitory interneurons without affecting the firing of excitatory neurons [106,107]. In addition, a targeted reduction in SCN2A expression using an ASO in a mouse model of Scn2a early seizure-onset DEE (Q/+ mice) substantially improved clinical outcomes. While untreated mice presented spontaneous seizures at P1 and did not survive beyond P30, the ASO-treated group showed a reduction in spontaneous seizures and a significantly extended life span [108].

Another therapeutic approach in current pharmacology is small molecules. They are synthesized compounds with low molecular weight (≤1000 Daltons) that can easily diffuse across cell membranes and modulate biological processes. They account for over 90% of marketed drugs, and their adaptability and efficacy make them a key element in treating a wide range of diseases, including cancer, infections, and neurological disorders [109,110]. GS-458967 (GS967) is an example of a small molecule that shows antiepileptic effects in a mouse model of DEE. It was originally reported to inhibit cardiac late sodium current (late INa) in order to suppress ventricular arrhythmias [111,112]. It reduced seizure frequency in Scn2a*^Q54^* mice and greatly improved their survival, along with inhibiting spontaneous action potential firing in pyramidal neurons isolated from Scn2a*^Q54^* mice [81]. Moreover, it suppressed spontaneous seizures and significantly improved the survival of Scn1a+/− mice [80]. Several other small molecules have been designed for Dravet syndrome, each with a distinct mechanism of action, including ataluren (a suppressant of premature stop codons; under clinical evaluation), soticlestat (a 24-hydroxylase cholesterol enzyme inhibitor), SPN-817 (an inhibitor of acetylcholinesterase), EPX-100, EPX-200, fenfluramine (serotonin modulators), and verapamil (a voltage-dependent calcium channel inhibitor) (comprehensive review in Miziak et al.) [113].

Leveraging the compensatory effects of voltage-gated ion channels represents a promising tool for the treatment of DEE. Currently, the treatment paradigm for DEE involves developing a specific drug for each genetically validated mutated gene, a process that can be time-consuming and costly and often leaves many patients without effective treatment options. However, an emerging and promising therapeutic strategy suggests that the same drug could potentially target a broader population of patients with mutations in different genes. This strategy is based on the understanding that mutations in different genes can lead to the same outcome: disruption in neuronal excitability and an impaired E/I ratio. Therefore, modulating other ion channels can potentially restore the brain’s homeostatic state by rebalancing the E/I ratio. This approach not only provides the potential for improved clinical outcomes, offering hope to a wider range of patients but also increases our understanding of the complex compensatory mechanisms underlying DEE. Moreover, it could simplify the drug development process, making it more efficient and cost-effective. It also opens up the possibility of repurposing existing drugs, which could accelerate the availability of new treatments.

In conclusion, continued research into these compensatory mechanisms and the manipulation of ion channel dynamics is crucial. This research could lead to the development of novel interventions that not only target the root causes of DEEs but also provide broader therapeutic options. Ultimately, this could significantly improve the quality of life for patients and their families, offering new hope and better management of this challenging group of disorders.

## Figures and Tables

**Table 1:** Summary of Selected Ion Channels.

Channel	Protein	Gene	Main DEE Phenotype	Main Functional Impact
**Sodium**	Nav1.1	SCN1A	DEE 6A (Dravet Syndrome)	LOF
	Nav1.2	SCN2A	DEE 11	GOF/LOF
	Nav1.6	SCN8A	DEE 13	GOF
**Potassium**	Kv1.1	KCNA1	DEE *	LOF
	Kv7.2	KCNQ2	DEE 7	LOF
	KNa1.1 (Slo2.2/Slack)	KCNT1	DEE14	GOF
**Calcium**	Cav2.1	CACNA1A	DEE 42	GOF/LOF
	Cav3.1	CACNA1G	DEE *	GOF

* While KCNA1 and CACNA1G are both identified as causal genes for DEE in the literature, the specific subtype of DEE associated with these genes is not well-defined. This is why we have listed them as “DEE” in the table without further specification.

## Data Availability

Not applicable.
